# TEMPO-Functionalized Carbon Nanotubes for Solid-Contact Ion-Selective Electrodes with Largely Improved Potential Reproducibility and Stability

**DOI:** 10.1021/acs.analchem.2c00395

**Published:** 2022-05-27

**Authors:** József Kozma, Soma Papp, Róbert E. Gyurcsányi

**Affiliations:** † Department of Inorganic and Analytical Chemistry, Budapest University of Technology and Economics, Műegyetem rkp. 3, H-1111 Budapest, Hungary; ‡ MTA-BME Lendület Chemical Nanosensors Research Group, Műegyetem rkp. 3, H-1111 Budapest, Hungary; § MTA-BME Computation Driven Chemistry Research Group, Műegyetem rkp. 3, H-1111 Budapest, Hungary

## Abstract

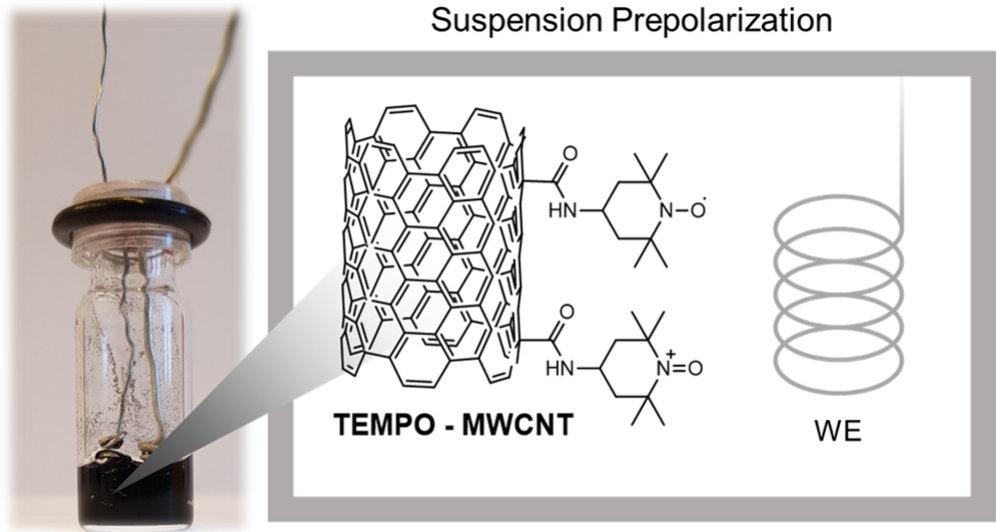

Solid-contact ion-selective electrodes (SCISEs) can overcome essential limitations of their counterparts based on liquid contacts. However, attaining a highly reproducible and predictable *E*^0^, especially between different fabrication batches, turned out to be difficult even with the most established solid-contact materials, i.e., conducting polymers and large-surface-area conducting materials (e.g., carbon nanotubes), that otherwise possess excellent potential stability. An appropriate batch-to-batch *E*^0^ reproducibility of SCISEs besides aiding the rapid quality control of the electrode manufacturing process is at the core of their “calibration-free” application, which is perhaps the last major challenge for their routine use as single-use “disposable” or wearable potentiometric sensors. Therefore, here, we propose a new class of solid-contact material based on the covalent functionalization of multiwalled carbon nanotubes (MWCNTs) with a chemically stable redox molecule, (2,2,6,6-tetramethylpiperidin-1-yl)oxyl (TEMPO). This material combines the advantages of (i) the large double-layer capacitance of MWCNT layers, (ii) the adjustable redox couple ratio provided by the TEMPO moiety, (iii) the covalent confinement of the redox couple, and (iv) the hydrophobicity of the components to achieve the potential reproducibility and stability for demanding applications. The TEMPO-MWCNT-based SC potassium ion-selective electrodes (K^+^-SCISEs) showed excellent analytical performance and potential stability with no sign of an aqueous layer formation beneath the ion-selective membrane nor sensitivity toward O_2_, CO_2_, and light. A major convenience of the fabrication procedure is the *E*^0^ adjustment of the K^+^-SCISEs by the polarization of the TEMPO-MWCNT suspension prior to its use as solid contact. While most *E*^0^ reproducibility studies are limited to a single fabrication batch of SCISEs, the use of prepolarized TEMPO-MWCNT resulted also in an outstanding batch-to-batch potential reproducibility. We were also able to overcome the hydration-related potential drifts for the use of SCISEs without prior conditioning and to feature application for accurate K^+^ measurements in undiluted blood serum.

## Introduction

Ion-selective electrodes (ISEs) with ionophore-based polymeric sensing membranes are at the core of monitoring the concentration of ions in the clinical, environmental, and process analysis fields. Currently, the development of ISEs largely shifted from fundamental mechanistic investigations and implementation of new ionophores toward application-based development of the existing ISEs. In this respect, their out-of-laboratory deployment that includes applications for wearable and disposable ion sensors is especially of perspective.^1−3^ Such applications call for low-cost, miniaturized, mass-produced ISEs with excellent potential stability, reliability, and little or no maintenance. Generally, these requirements can be best met by replacing the classical symmetrical liquid contact (LC)-based configuration employing an internal solution with a solid contact (SC).^4^

Containing the inner solution in a hydrogel matrix represented an ingenious intermediate solution as it preserved the well-defined inner boundary potential of the liquid contact electrodes while enabling their mass production by automated drop-casting.^5^ Still the small volume of the initially dry hydrogel posed difficulties for their immediate use because upon placing them in an aqueous solution, the transmembrane water uptake led to the swelling of the hydrogel and consequently to a significant potential drift. The poor attachment of the ion-selective membranes to the hydrogel and the establishment of the osmotic balance in the sample posed additional difficulties. While all of these issues were solved in clinical blood electrolyte analyzers achieving excellent analytical performances using flow-through systems to mechanically secure the respective ISEs and regularly calibrate them,^6,7^ their application as a disposable single-use or wearable sensor is by far not trivial. Therefore, these applications remain an important niche for solid-contact ISEs (SCISEs), which are expected to provide a similarly stable phase boundary potential of the inner membrane interface as their LC counterparts and at the same time facilitate and simplify the mass production of miniaturized ISEs.

Owing to significant advances in solid-contact materials, state-of-the-art solid-contact ISEs (SCISEs) at present largely reached this goal.^8^ An extremely broad range of materials were implemented including various conducting polymers,^9−16^ carbon black,^17^ intercalation compounds,^18^ carbon nanotubes,^19,20^ graphene,^21^ nanostructured conducting materials,^22−25^ and redox molecules.^26−28^ However, controlling and reproducing the standard potentials (*E*^0^) of SCISEs that is straightforward for LCISEs remained challenging. While calibration solves this problem for laboratory applications, for single-use, disposable sensors as well as for wearable sensors, the individual calibration of each electrode may not be possible and especially not practical. Thus, the development of so-called “calibration-free” SCISEs,^29^ with appropriate electrode-to-electrode reproducibility, contours as perhaps the greatest challenge before mass-produced SCISEs will be commercially available for everyday use in wearable, disposable, and field-deployed analytical devices.^29,30^ By calibration-free sensors, we mean calibration of a small, but representative number of electrodes and the extrapolation of these calibration parameters for large fabrication batches. To achieve this goal, the SCISEs should feature highly reproducible calibration parameters and while the slopes are generally close to the theoretical Nernstian value, the *E*^0^ of SCISEs may vary tens or even hundreds of mVs. Since for a singly charged analyte ion 1 mV error in the measured potential of an ISE with theoretical response translate into an error of 4% in concentration, the importance of the *E*^0^ reproducibility becomes obvious. Accordingly, it received lately major attention^29^ for the main types of the potential stabilizing mechanism of the solid-contact materials, i.e., redox (conducting polymers, redox polymers/materials/molecules) and capacitive (large-surface-area conductors, e.g., carbon nanotubes, graphene, etc.).

The quest for suitable materials was complemented by methodological approaches that generally involve the application of an external potential^11,31^ or short-circuiting^32^ to adjust the *E*^0^, i.e., to drive externally the potential of a large number of SCISEs connected together in solution toward the same potential. While such procedures were shown to be applicable for both capacitive and redox-type solid contacts,^33^ they are suited only to compensate for small differences (a few mVs) in the *E*^0^ values after SCISE fabrication. Moreover, the potential value to which the SCISEs are polarized is critical, i.e., if it falls far from the equilibrium potential of the electrodes, it will lead to a very temporary improvement followed by drifting and diverging potential responses.

The exact origin of the *E*^0^ differences of SCISEs made with the exact same fabrication procedure and materials is not known, but it is likely to be caused by small chemical, geometrical, and morphological differences/heterogeneities of the respective SCISEs including variations in the redox state and surface functionality of SC materials, as well as the degree of interpenetration between the ion-selective membrane and SC.^34^ Given the uncertainties in most industrial manufacturing procedures, some sort of adjustment to level small differences may be needed for most mass fabricated SCISEs. Besides calibration-free applications, the reproducibility and even adjustment of *E*^0^ values of large fabrication batches is beneficial also in terms of quality control. However, it is rather unpractical to apply potential leveling for fully prepared SCISEs, i.e., to contact all electrodes and place them in an electrolyte solution. It would be much more convenient if a “potential preadjusted” solid contact is applied on the substrate electrode during fabrication that largely devoids further actions with the fabricated SCISEs. In principle, this is possible with redox-active SC materials by adjusting the ratio of the oxidized and reduced forms. However, this is not trivial with conducting polymers that show a broad continuum of redox potentials^35,36^ rather than well-defined redox peaks, while it is also desired to avoid extraction of molecular redox couples into the ion-selective membrane that may affect their selectivity. These requirements may be best addressed by covalent confinement of redox molecules to a solid support as we proposed recently by covalent functionalization of MWCNTs with ferrocene groups.^37^ However, for long-term potential stability, an extremely stable and reversible redox functionality is required that is not susceptible to light and oxidation by molecular oxygen,^38^ along with ensuring the appropriate hydrophobicity of the SC materials.

Here, we introduce (2,2,6,6-tetramethylpiperidin-1-yl)oxyl (TEMPO)-functionalized multiwalled carbon nanotubes (TEMPO-MWCNTs) as a novel solid contact material for the fabrication of ion-selective electrodes with highly reproducible standard potential (*E*^0^). Covalent grafting of TEMPO to MWCNTs combines the advantages of both redox and high-capacitance materials but prevents the leaching of the redox-active compound from the solid contact layer into the ion-selective membrane (ISM). In terms of chemical stability,^39^ as well as pH,^40^ light, and oxygen insensitivity,^41^ TEMPO outperforms many other redox-active compounds (e.g., ferrocene^42^), making it an “ideal” candidate for the redox functionalization of MWCNTs. Here, we explore the feasibility of using TEMPO-MWCNT as solid contact in cation-selective electrodes by addressing the batch-to-batch potential reproducibility of SCISEs. In this respect, we investigate the preadjustment of the potential of TEMPO-MWCNT in suspension prior to their drop-cast on the substrate transducer, which could be a major benefit and convenience in terms of large-scale SCISE fabrication.

## Experimental Section

### Chemicals and Materials

High-molecular-weight PVC (HMW PVC), potassium ionophore I (Valinomycin), bis(2-ethylhexyl) sebacate (DOS), tetrahydrofuran (THF) (all selectophore grade), anhydrous acetonitrile (ACN, 99.5%), anhydrous dimethylformamide (DMF, 99.8%), sulfuric acid (H_2_SO_4_, ACS reagent, 95–98%), *N*-(3-dimethylaminopropyl)-*N*′-ethylcarbodiimide hydrochloride (EDC, 98%), *N*-hydroxysuccinimide (NHS), 4-amino-2,2,6,6-tetramethylpiperidine-1-oxyl (4-amino-TEMPO, free radical), silver nitrate (AgNO_3_), and tetraethylammonium nitrate (TEANO_3_) were purchased from Sigma-Aldrich. Tetrabutylammonium hexafluorophosphate (TBAPF_6_, for electrochemical analysis, ≥99%) and nitric acid (≥69%) were obtained from Fluka. Potassium tetrakis(pentafluorophenyl)borate (KTFAB, 97%) was received from Alfa Aesar. Multiwalled carbon nanotubes (MWCNT) were purchased from HeJi, Inc. (30–50 nm diameter, 0.5–200 μm length, M4905). Human serum was received from HyTest Ltd. (Lot. 15/05-8TFS) and Merck Millipore (Lot. 3733758). Absolute ethanol and methanol (AR grade) were obtained from Molar Chemicals Ltd. (Halásztelek, Hungary). The aqueous solutions were prepared with deionized water (DIW) with a resistivity of 18.2 MΩ cm.

### Synthesis of TEMPO-Functionalized MWCNTs (TEMPO-MWCNT)

**Figure 1:**
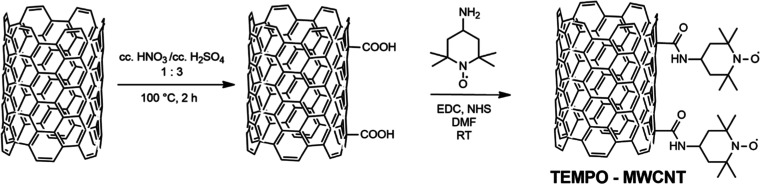
Schematic of the chemical functionalization of MWCNTs with TEMPO group.

For the synthesis of TEMPO-functionalized MWCNTs (Figure 1), we adapted a previously reported procedure^43^ changing the MWCNT preactivation step. Briefly, 0.4 g of pristine MWCNTs was oxidized to form carboxylic surface functionalities by stirring in cc. HNO_3_/cc. H_2_SO_4_ (3:1 (V/V %), 32 mL) at 100 °C for 2 h. The suspension was cooled to room temperature and added to deionized water (350 mL). The carboxylated MWCNTs (MWCNT-COOH) were isolated after three repeated sequences of centrifugation (7000 rcf, 30 min), supernatant removal, and DIW washing. The aqueous suspension was dried at 60 °C overnight, resulting in 230 mg of MWCNT-COOH. MWCNT-COOH (30 mg) was sonicated in 5 mL of dry dimethylformamide for 5 min, and then the carboxyl groups were activated by excess amounts of EDC (7.8 mg) and NHS (5.2 mg). The suspension was stirred at room temperature for 2 h, and then 4-amino-2,2,6,6-tetramethylpiperidine-1-oxyl free radical (12.9 mg, dissolved in 1.5 mL of dry DMF) was added. The suspension was stirred for another 72 h at room temperature, and then the TEMPO-MWCNTs were separated by centrifugation at 25 000 rcf (for every 1 mL of the DMF suspension, 1 mL of methanol was added for adequate separation). The product was washed four times with methanol and dried at 60 °C for 12 h to obtain 21.7 mg of TEMPO-MWCNTs. The dried synthetic batches of TEMPO-MWCNTs were stored under ambient conditions with no special precautions.

### K^+^-Selective SCISE Fabrication

Glassy carbon (GC) electrodes (⌀1.6 mm, incorporated in a polyether ether ketone (PEEK) body with an outer diameter of 6.0 mm; Bio-Logic Science Instruments) were polished with 0.3 μm alumina suspension and then rinsed with ethanol and water. The electrodes were sonicated for 5 min in DIW, then rinsed again with DIW and ethanol, and finally dried under N_2_ stream. Potassium ion-selective membranes (K^+^-ISM) were prepared with the following composition: 32.9% (w/w) HMW PVC, 65.7% DOS, 1.06% valinomycin, and 0.34% KTFAB. The components were dissolved in THF to prepare an ISM cocktail with 20% dry weight. TEMPO-MWCNTs were accurately weighed in and sonicated in dry THF for 20 min to produce a 5 mg/mL suspension. Such suspensions were always freshly made before electrode preparations to devoid concentration changes due to solvent evaporation. This suspension (25 μL) was drop-cast onto the glassy carbon electrode confining it with a custom-made polypropylene (PP) mask to the conductive GC surface (Scheme S1). The PP mask was removed after the MWCNT layer dried, and 40 μL of the ISM cocktail was deposited onto the MWCNT-SC layer in two consecutive steps (2 × 20 μL) and allowed to dry overnight, which resulted in ca. 220 μm thick ISMs. Coated-wire ion-selective electrodes were made following the same procedure but without the solid-contact layer deposition.

### Potentiometric Measurements

Potentiometric measurements were conducted in stirred solutions using a 16-channel high-input impedance voltmeter (10^15^ Ω, Lawson Laboratories, Malvern, PA), and the reference electrode in aqueous solutions was a Ag|AgCl|3 M KCl||1 M LiOAc double-junction electrode (no. 6.0729.100, Metrohm AG). The potential stability of TEMPO-MWCNT-based SCISEs was monitored in a 0.01 M KCl solution. The gas (O_2_ and CO_2_) sensitivity of the TEMPO-MWCNT-based SCISEs was studied by bubbling air, CO_2_ (generated from dry ice in a balloon), or Ar through a solution of 0.01 M KCl. The light sensitivity of the TEMPO-MWCNT-SCISEs was also determined in 0.01 M KCl by switching illumination: room light (20 min), darkness (20 min), intense cold light (20 min; 12 W, 850 lumen). Potentiometric selectivity coefficients were determined with the separate solution method in 0.1, 0.01, and 0.001 M chloride salts of K^+^, and the interfering ions Li^+^, Na^+^, NH_4_^+^, and Mg^2+^. The potentiometric aqueous layer test was done with fully conditioned K^+^-SCISEs by changing the solution from 0.1 M KCl to 0.1 M NaCl, and then back to 0.1 M KCl.

### Potentiostatic/Galvanostatic Measurements

Cyclic voltammetry, electrochemical impedance spectroscopy (EIS), and chronopotentiometric measurements were done with a Reference 600 Potentiostat/Galvanostat (Gamry Instruments, Warminster, PA) using a three-electrode cell. The cyclic voltammetric curves of the TEMPO-MWCNT layers were measured in 0.1 M TBAPF_6_ in ACN with different scan rates (25–200 mVs^–1^), using a Pt wire and Ag|0.01 M AgNO_3_||0.1 M TEANO_3_ as counter and reference electrodes, respectively. The impedance spectra of TEMPO-MWCNT layers were recorded in 0.1 M TBAPF_6_ in ACN within the frequency range of 1000 kHz to 10 mHz, at open-circuit potential with 20 mV AC amplitude (rms) (using Ag wire as a quasi-reference and Pt wire as a counter electrode). The chronopotentiometric measurements of the TEMPO-MWCNT-based SCISEs were performed in 0.01 M KCl by applying +1 nA for 60 s and then −1 nA for 60 s. In this case, Ag/AgCl wire was used as the reference electrode and Pt wire as the counter electrode.

## Results and Discussion

### Characterization of the TEMPO-MWCNT Solid Contact

**Figure 2:**
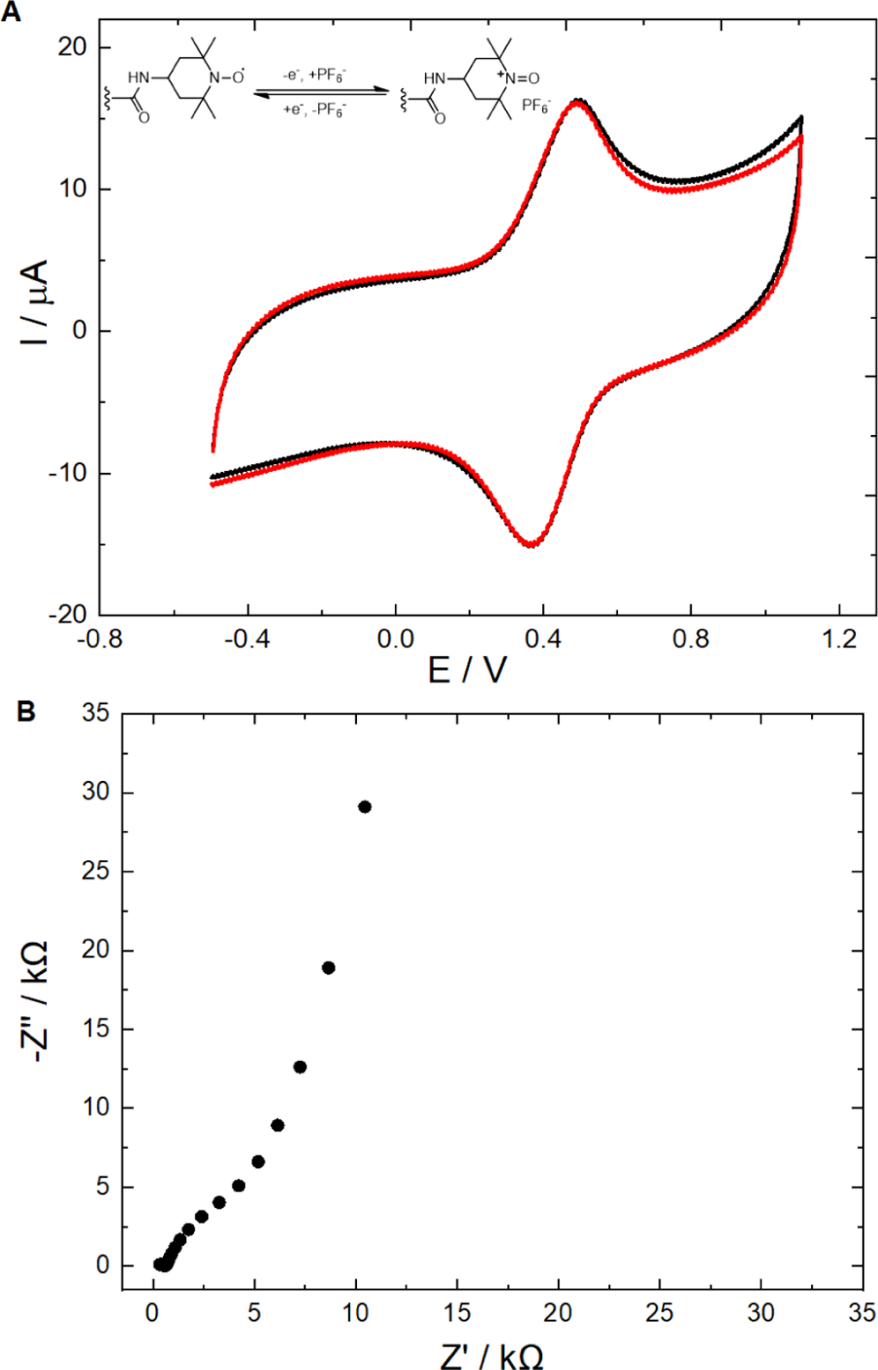
(A) Cyclic voltammograms of the TEMPO-MWCNT layer measured in 0.1 M TBAPF_6_ in acetonitrile. Inset: Redox reaction of the TEMPO group.^45,46^ (B) Electrochemical impedance spectrum of the TEMPO-MWCNT layer in 0.1 M TBAPF_6_ (ACN). The TEMPO-MWCNT layer was made by drop-casting 20 μL of 5 mg/mL TEMPO-MWCNT suspension in THF onto GC electrodes.

The cyclic voltammetric curves (CVs) of the TEMPO-MWCNT modified glassy carbon electrodes (Figure 2A) in 0.1 M TBAPF_6_ in acetonitrile revealed a pair of well-defined redox peaks centered around 0.42 V, attributed to the reversible oxidation of the TEMPO groups (Figure 2A, inset), superposed on the large capacitive current background of the MWCNT layer. Repeated cycles showed no change in the recorded CVs, confirming the covalent attachment of the TEMPO and the overall stability of the drop-cast TEMPO-MWCNT layer on the electrode surface. The peak currents were found to be proportional to the square root of the scan rate (Figure S1), indicating that the current is limited by the diffusion of the counter ion accompanying the redox reaction of the TEMPO group, which is in a good agreement with previous findings.^43^

The capacitance of the solid contact, i.e., of the TEMPO-MWCNT layer on GC electrodes, was investigated by EIS (Figure 2B) as a high capacitance has also a potential stabilizing effect as shown previously for purely capacitive CNT-based SCISEs.^44^ The areal capacitance was calculated from the impedance values in the low-frequency (10–100 mHz) part of the EIS spectrum, where the capacitive behavior is dominant. We found that the areal capacitance (29.7 mF cm^–2^) improved considerably compared to other modified MWCNTs such as octadecylamine-modified MWCNTs (7.66 mF cm^–2^)^33^ and ferrocene-functionalized MWCNTs (12.33 mF cm^–2^).^37^ Chronopotentiometric reverse pulse experiments^9^ (Figure S2) confirmed the high capacitance proving also the very low polarizability of the TEMPO-MWCNT-based SCISEs (0.8 mV min^–1^) compared to “coated-wire” electrodes (229.8 mV min^–1^), using the very same composition K^+^-selective membrane.

### Potential Reproducibility and Stability of TEMPO-MWCNT-Based SCISEs

**Figure 3:**
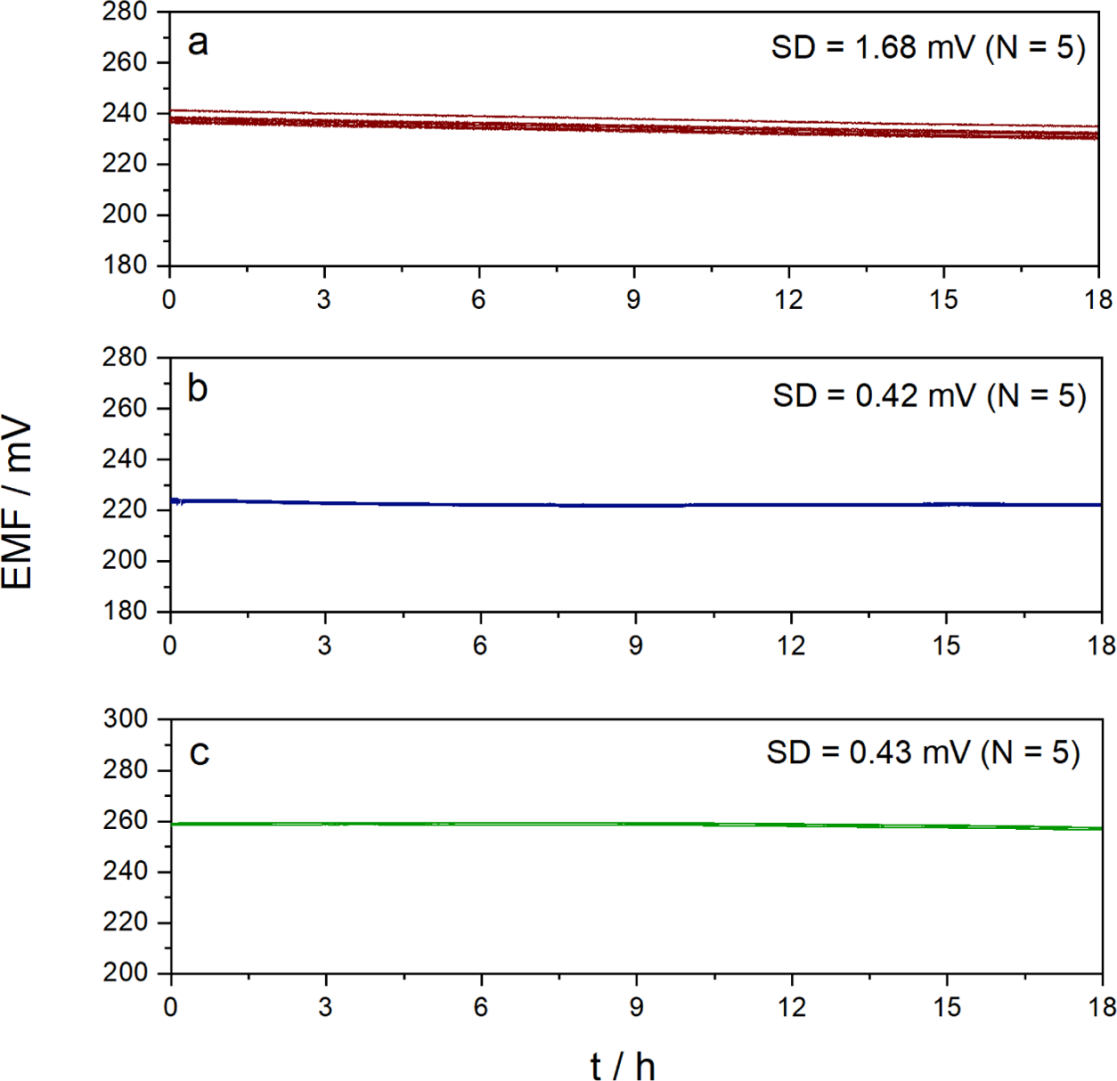
Potential stability of the TEMPO-MWCNT-based K^+^-SCISEs: (a) without any pretreatment; (b) after short-circuiting them for 48 h in 10 mM KCl; (c) prepared from a TEMPO-MWCNT suspension prepolarized at 75 mV for 20 min. For rigorous comparison with the short-circuited electrodes, the potential curves are plotted for all batches after a 48 h conditioning in 10 mM KCl solution.

We compared the potential reproducibility and stability of three batches of TEMPO-MWCNT-based SCISEs: without any pretreatment, 48 h short circuit, and with prepolarized MWCNT-TEMPO suspension as the solid contact. For a rigorous side-by-side comparison, since the short-circuited electrodes had to be inherently kept in 10 mM KCl for 48 h, the other two types of electrode batches were also placed in 10 mM KCl for 48 h, i.e., the onset of the measurements in Figure 3 (*t* = 0) follows this 48 h interval. We found that the potential reproducibility of the TEMPO-MWCNT-based SCISEs was already remarkably good (SD = 1.68 mV, *n* = 5) (Figure 3a) without applying any special pretreatment (except conditioning). For comparison, SCISEs based on state-of-the-art purely capacitive octadecyl-modified MWCNT (OD-MWCNT)-based solid contacts had SD values of ca. 4–5 mV without pretreatment.^33^ Similar results were reported for MWCNT-based SCISEs^47^ and other carbon-based nanomaterials such as colloid-imprinted mesoporous carbon^48^ and ordered mesoporous carbon spheres.^49^ Thus, the redox functionalization of carbon nanotubes is clearly beneficial compared with purely capacitive contacts and that applies also for the short-term potential stability that improved from 980 ± 600 μV/h (OD-MWCNT) to −335 ± 7 μV/h for TEMPO-MWCNT. Short-circuiting the K^+^-SCISEs during conditioning in KCl solution is an effective way to further improve the potential reproducibility, and if necessary, this can be made after fabrication and before packaging. Figure 3b shows that following a 48 h short-circuiting, the SD of the potential was 0.42 mV (*n* = 5; with a potential stability of −133 ± 8 μV/h). The potential reproducibility is among the best reported values so far^8,29^ and outperforms attempts with other modified CNT-based solid contacts, e.g., OD-MWCNT (SD = 1.56 mV)^33^ and Fc-MWCNT (SD = 1.85 mV).^37^ However, despite the excellent results, as stated earlier, the application of any post-treatment to the SCISEs is inconvenient and costly fabrication-wise. Therefore, we explored the leveling of SCISEs to the same *E*^0^ by polarizing the continuously stirred TEMPO-MWCNT suspension for 20 min at 75 mV, which is the “equilibrium” potential determined in the same suspension before the polarization, using a two-electrode cell with large-surface-area Pt coil and Ag coil serving as working and quasi-reference electrodes, respectively. The K^+^-SCISEs prepared by drop-casting the prepolarized TEMPO-MWCNT possessed practically the same reproducibility (SD = 0.43 mV, *n* = 5) (Figure 3c) as the short-circuited SCISEs, and furthermore, the potential stability was significantly improved (−70 ± 7 μV/h).

**Figure 4:**
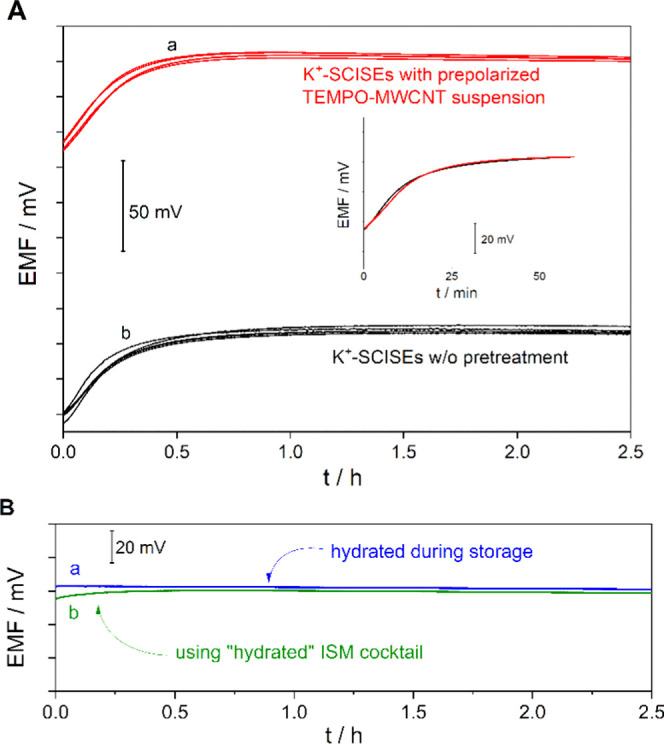
(A) Potential transients of TEMPO-MWCNT-based K^+^-SCISEs recorded at their first contact with an aqueous solution (10 mM KCl): (a) prepared from a TEMPO-MWCNT suspension prepolarized at 75 mV and (b) without any pretreatment. The electrode potentials of the batch with prepolarized suspension were shifted with a constant value of +100 mV to facilitate the separate assessment of the transients of the two different electrode batches. The inset shows the reproducibility of the initial potential drift by comparing the potential transients of the same electrode at first contact with the 10 mM KCl solution (red) and after drying the membrane under ambient conditions for 48 h (black). (B) Potential measurements with prehydrated electrode membranes during storage (a) and by additionally adding 5% w/w water into the drop-cast membrane cocktail (b).

Of note, such performance needs conditioning of the SCISEs because at first contact with a solution, the hydration of the dry membrane^50^ results in a positive potential drift of ca. 50 mV in 60 min (Figure 4A). The threshold potential drift of 0.3 mV/min reported earlier for highly hydrophobic PEDOT derivative-based SC to determine the equilibration time at first contact with an aqueous solution,^16^ however, was reached in ca. 25 min. Very importantly, we found that even during the drift, the excellent potential reproducibility was preserved and the drift could be rigorously reproduced during drying–hydration cycles of the ion-selective membrane (Figure 4A, inset). In principle, the asymmetric configuration of the SCISEs makes them generally prone to hydration-related potential drift. This phenomenon is largely overlooked despite being clearly a limitation for applications requiring out-of-shelf, immediate use of SCISEs without any conditioning. The very few studies that addressed the initial potential drift of SCISEs tried to alleviate this problem through the adjustment of the SC and membrane layer thickness as shown by Guzinski et al.^51^ or additionally using a special flow-through cell,^14^ but IS membranes less prone to water uptake^52^ or very thin IS membranes^53^ may also lead to a faster potential stabilization. Here, we investigated as a more general concept the prehydration of the ISMs during their preparation and/or storage. In the first case, 0.5% w/w DIW was added in the THF-formulated membrane cocktail and drop-cast on the SC (the water content was chosen based on the maximal water uptake reported for plasticized PCV membranes^54^). In the second case, 10 μL of DIW was dropped onto the IS membrane surface. In both cases, the electrodes were sealed using a 3D-printed cylindrical enclosure to avoid the drying of the ISM during storage. Since planar sensors, e.g., glucose sensors, are often commercialized individually sealed in an aluminum foil blister, both procedures are, in principle, compatible with packaging technologies. The preliminary results of using such prehydrated electrodes (stored for at least 24 h) right after removing the sealing, without any conditioning, were very promising, i.e., the initial drift of the unconditioned electrodes (ca. 54 mV/h) was reduced dramatically to −0.4 and 4.0 mV/h for electrodes stored with a drop of DIW and adding DIW to the membrane cocktail, respectively. It is remarkable that the prehydration can be done with pure water and these simple procedures lead to electrodes that are immediately operational and with reproducible potentials (SD = 1.26 mV, *n* = 4).

**Figure 5:**
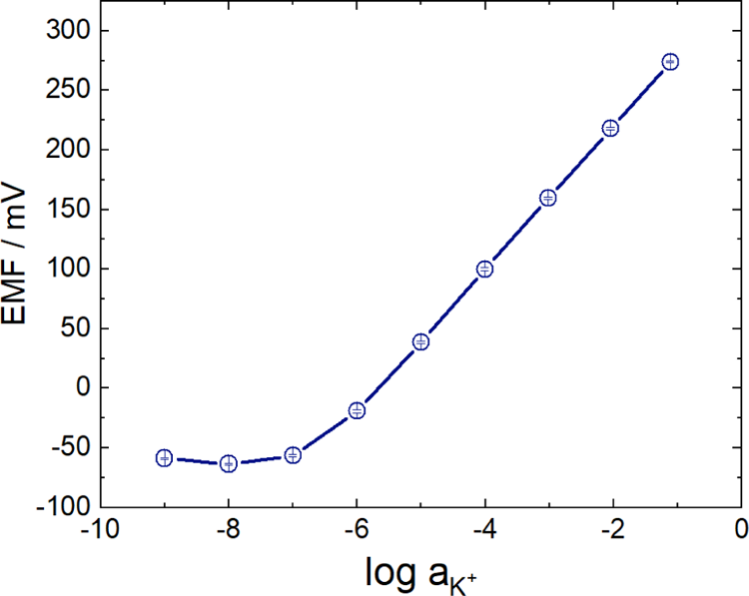
Calibration curve of TEMPO-MWCNT-based K^+^-SCISEs (*n* = 6). The electrodes were short-circuited for 48 h in 0.01 M KCl solution before calibration.

The calibration of the SCISEs revealed reproducible Nernstian slopes down to 10^–6^ M and a limit of detection of ca. 2.4 × 10^–7^ M. Remarkably, as shown in Figure 5 for short-circuited K^+^-SCISEs, the potential reproducibility proved to be excellent throughout the whole concentration range (10^–9^–10^–1^ M). Since most often there is a marked potential divergence of SCISEs for the low concentration range, this is an additional indication of the high level of control over SCISE fabrication that can be attained using TEMPO-MWCNT as solid contact.

**Figure 6:**
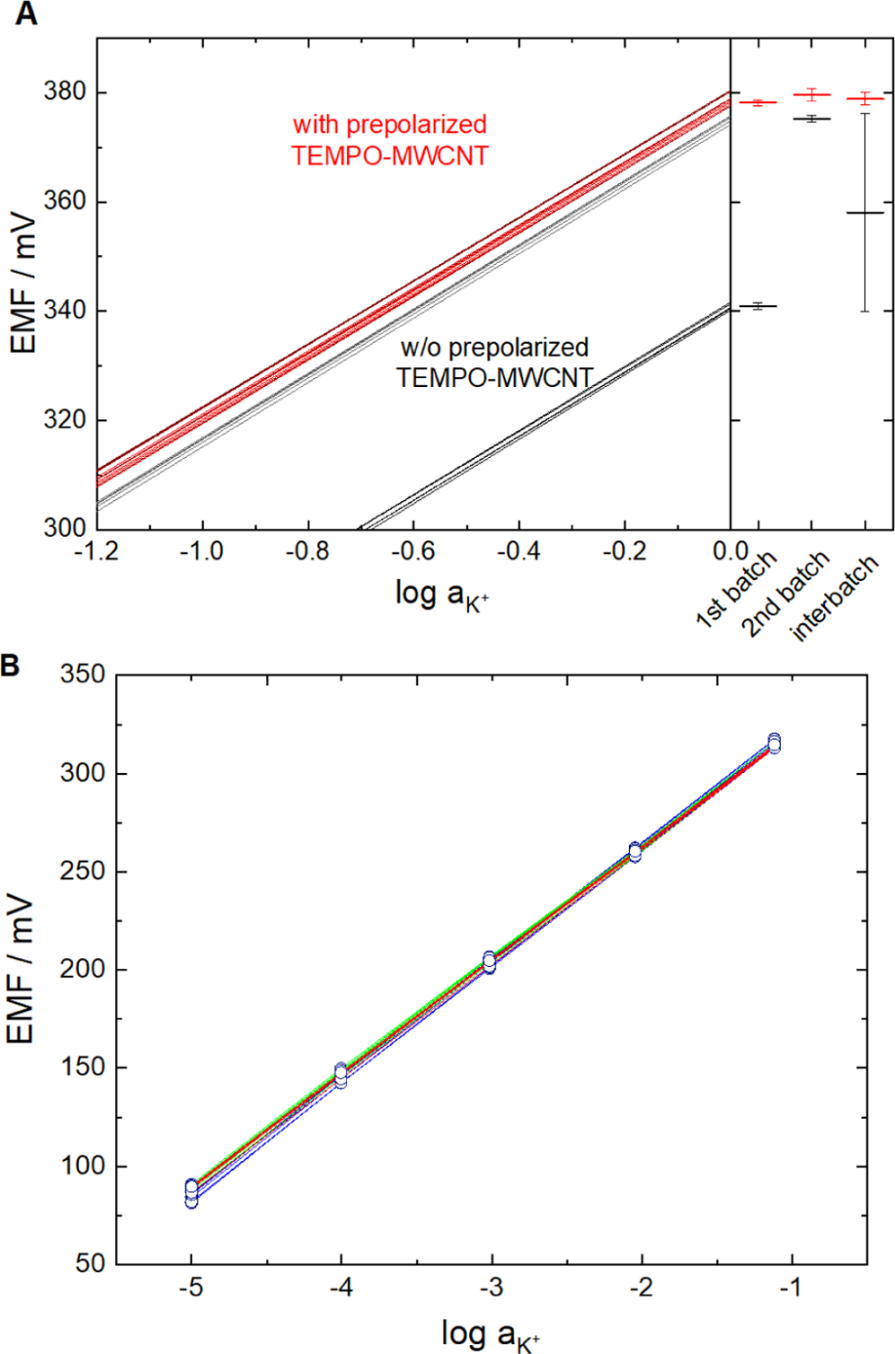
(A) Left: Extrapolated potential values (the intercept is indicative of their *E*^0^) of two parallel batches of K^+^-SCISEs based on TEMPO-MWCNT solid contact with prepolarization (red) and w/o prepolarization (black). Right: Corresponding average *E*^0^ values and their standard deviation for the individual batches of TEMPO-MWCNT-based K^+^-SCISEs and interbatch values. (B) Calibration curves of three batches of 6 K^+^-SCISEs each (*n* = 18) based on drop-casting the prepolarized TEMPO-MWCNT suspension. The time span between the preparation of the first and last batch was ca. 1 year.

However, it is rather common to obtain a good *E*^0^ reproducibility for a single fabrication batch consisting of a small number of electrodes. It is more challenging, as emphasized recently,^29^ to secure the same reproducibility for multiple fabrication batches separated by longer intervals. Despite strictly following the same fabrication protocols, most often significant changes in the *E*^0^ values are observed for the different electrode batches (often hidden by relative potential scales). Therefore, we investigated whether the well-defined potential adjustment possibility offered by redox solid contacts can be exploited to address the interbatch reproducibility issue. We compared the *E*^0^ and the slope of the calibration curve for two parallel batches of TEMPO-MWCNT-based K^+^-SCISEs: (i) with 48 h short-circuiting, but without prepolarization and (ii) using the prepolarized TEMPO-MWCNT suspension (Figure 6A). In the case of the short-circuited ISEs, both batches (*n* = 5) had excellent single-batch *E*^0^ reproducibility (SD = 0.6–0.7 mV), but the average *E*^0^ values of the two batches differed drastically (ca. 30 mV) (Table S1), resulting ultimately in an extremely large interbatch SD of 18 mV (*n* = 10). However, using TEMPO-MWCNT suspensions prepolarized at the same potential for the SCISE preparation, i.e., setting the ratio of the oxidized and reduced forms to the same value, can theoretically address this issue. Two batches of 6 K^+^-SCISE each were prepared with a week interval using prepolarized TEMPO-MWCNT suspensions to test this assumption. The difference in their average *E*^0^ value was only 1.5 mV, which is a spectacular improvement with respect to the electrodes that were made without adjusting the potential of the redox-functionalized CNT suspension (30 mV).

To expand the time frame between electrode batches, a new set of electrodes (*n* = 6) was fabricated using the same procedure ca. a year later. Of note, the potential of the TEMPO-MWCNT was within 5 mV in all batches compared to the initial value of 75 mV, chosen for prepolarization. Comparing the potentiometric responses with the earlier batches revealed practically the same Nernstian response for all of the 18 electrodes, as shown in Figure 6B, with an SD of 2.13 mV for the potential response (Table 1). This supports the efficiency of TEMPO-MWCNT suspension prepolarization in improving the potential reproducibility of the relevant SCISEs and shows the prospective of this approach for their convenient and reliable preparation with excellent batch-to-batch potential reproducibility.

**Table 1:** Calibration Parameters of Three Batches of 6 K^+^-SCISEs Each Made Using Suspension Polarized TEMPO-MWCNT as Solid Contact

	1st batch (*n* = 6)		2nd batch (*n* = 6) after 1 week		3rd batch (*n* = 6) after ca. 1 year		for all 18 electrodes	
	average	SD	average	SD	average	SD	average	SD
*E*^0^ (mV)	378.2	0.57	379.7	1.15	382.2	1.94	380.0	2.13
slope (mV/dec)	58.0	0.1	57.8	0.2	59.5	0.1	58.4	0.8

Investigating the medium-term stability of the electrodes (1 week), we found that the K^+^-SCISE prepared using prepolarized TEMPO-MWCNT suspension again outperformed their counterparts made without prepolarization, but using short-circuiting. The standard deviation of the potential was 0.79 mV at the end of 1-week continuous measurement, which is insignificantly larger than 0.71 mV registered at start.

### Potentiometric Water Layer, Gas and Light Sensitivity Tests

**Figure 7:**
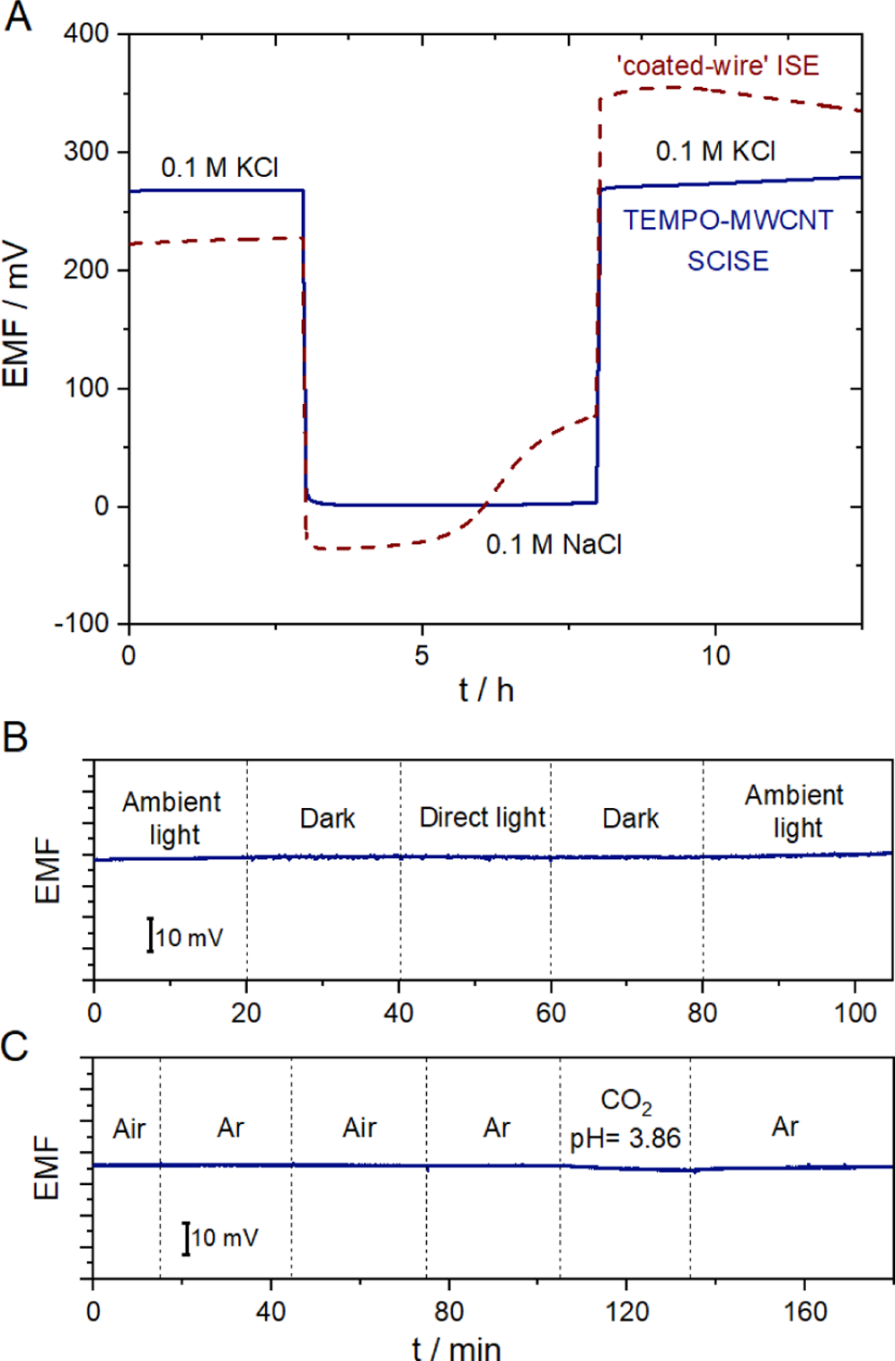
(A) Potentiometric aqueous layer test of TEMPO-MWCNT-based K^+^-SCISEs (solid line) and a simple coated-wire K^+^-ISE (dashed line). (B) Light sensitivity and (C) CO_2_ and O_2_ sensitivity of TEMPO-MWCNT-based K^+^-SCISEs measured in 0.01 M KCl (stirred solution). RE: Ag|AgCl|3 M KCl||1 M LiOAc.

The excellent reproducibility and consistency of the potential responses determined in ambient laboratory conditions made very unlikely that the prepared electrodes are susceptible to the formation of an aqueous layer beneath the ion-selective membrane nor that they are sensitive to light fluctuations and ambient gases that readily permeate through the ion-selective membrane. Indeed, the potentiometric water layer test^55^ of the fully conditioned TEMPO-MWCNT-based K^+^-SCISEs revealed no evidence of an aqueous layer formation (Figure 7A) as shown by the staircase-type potential responses as a result of changing back and forth between primary (0.1 M KCl) and interfering ion (0.1 M NaCl) solutions. Of note, the logarithmic selectivity coefficients (Table S2) of the TEMPO-MWCNT-based K^+^ SCISEs for the most relevant interfering ions in biological samples were found to be in agreement with the expected values.^37^ However, the coated-wire electrodes used as control (measured in parallel under the same solutions with TEMPO-MWCNT-based K^+^-SCISEs) showed the characteristic potential drifts of an electrode with an aqueous layer beneath the ISM.

The lack of light sensitivity (photovoltaic effect) of various carbon nanotube-based electrodes is well documented. However, owing to the redox functionalization of the MWCNTs, the respective K^+^-SCISEs were tested by exposing the electrodes to various light intensities. The qualitative measurements revealed no potential changes whatsoever due to light intensity changes (Figure 7B). The effect of the main ambient gases (O_2_ and CO_2_) was also tested by bubbling air and CO_2_ sequentially in 0.01 M KCl separated by Ar flushing steps. Figure 7C clearly indicates no significant change in the potential even after drastic changes in the dissolved gas concentrations.

## Conclusions

Overall, the TEMPO-MWCNT-based K^+^-SCISEs due to the inherent potential stabilizing effect of the redox groups grafted on the carbon nanotubes had an excellent within-batch potential reproducibility (SD = 1.68 mV), which was further improved by short-circuiting the electrodes with each other (SD = 0.42 mV, *n* = 5) for 48 h or, most appealingly, by adjusting the potential of the TEMPO-MWCNT suspension prior to electrode fabrication (SD = 0.43 mV, *n* = 5). While short-circuiting the electrodes together is efficient in leveling small potential differences, it can be applied for a fabrication batch and it is rather inconvenient, i.e., the electrodes need to be placed into a solution, electrically contacted, and then washed before packaging. However, the prepolarization of the TEMPO-MWCNT suspension prior to its use for electrode fabrication can be applied for different fabrication batches and results in a remarkable reproducibility of the respective K^+^-SCISEs. Therefore, taking advantage of the inherent capacitive and redox potential stabilizing mechanisms of TEMPO-MWCNT solid contacts, and covalent confinement of the redox moiety appears to be a feasible approach for convenient, large-scale fabrication of solid-contact ion-selective electrodes with state-of-the-art analytical performance and outstanding potential reproducibility, which are important prerequisites for their calibration-free application as single-use or wearable sensors. In this respect, we show preliminary results in terms of reducing the membrane hydration-related initial potential drift of unconditioned SCISEs by prehydrating the membranes during preparation and/or storage as well as calibration-free measurements of K^+^ concentration in blood serum revealing competitive accuracy (see the Supporting Information).

## Supporting Information Available

TEMPO-MWCNT SCISE fabrication process, TEMPO-MWCNT solid-contact CV characterization, TEMPO-MWCNT SCISE chronopotentiograms, selectivity coefficients, potential reproducibility (short-circuited), and blood serum measurements (experimental details, calibration curves, measurement curves) (PDF)
