# Supplementary material for: TEMPO-Functionalized Carbon Nanotubes for Solid-Contact Ion-Selective Electrodes with Largely Improved Potential Reproducibility and Stability
Source: Anal Chem. 2022 May 27;94(23):8249–57. doi: 10.1021/acs.analchem.2c00395 (PMC9201804; doi:10.1021/acs.analchem.2c00395)
Supplement: Supplementary file 1 — ac2c00395_si_001.pdf [file ac2c00395_si_001.pdf]

Supporting information

TEMPO-functionalized carbon nanotubes for solid contact ion-selective electrodes with largely improved potential reproducibility and stability

József Kozma[a,b], *Soma Papp*[a,b], *Róbert E. Gyurcsányi*[a,b,c]*

[a]Department of Inorganic and Analytical Chemistry, Budapest University of Technology and Economics, Műegyetem rkp. 3, H-1111 Budapest, Hungary, E-mail: gyurcsanyi.robert@vbk.bme.hu
[b]MTA-BME Lendület Chemical Nanosensors Research Group, Műegyetem rkp. 3, H-1111 Budapest, Hungary
[c]MTA-BME Computation Driven Chemistry Research Group, Műegyetem rkp. 3, H-1111 Budapest, Hungary

*Corresponding author: gyurcsanyi.robert@vbk.bme.hu

Table of contents:

- Fabrication scheme of the TEMPO-MWCNT based K^+-SCISE
- Characterization of the TEMPO-MWCNT solid contact with CV
- Chronopotentiometric characterization of TEMPO-MWCNT based K^+-SCISE and coated-wire ISE
- Potential reproducibility of TEMPO-MWCNT-based K^+-SCISEs after 48 hour-long short-circuiting for two different batches
- Potentiometric selectivity coefficients for TEMPO-MWCNT-based K^+-SCISEs
- Measurements of K^+ concentration in blood serum
- Calibration curves for K^+ with TEMPO-MWCNT-based SCISEs in serum-like standard solutions, potential traces measured with TEMPO-MWCNT-based SCISEs in undiluted serum sample
- The composition of standard solutions for calibration during serum measurements
- "Calibration-free" measurements of K^+ concentration in serum

Fabrication of TEMPO-MWCNT based K^+-SCISEs

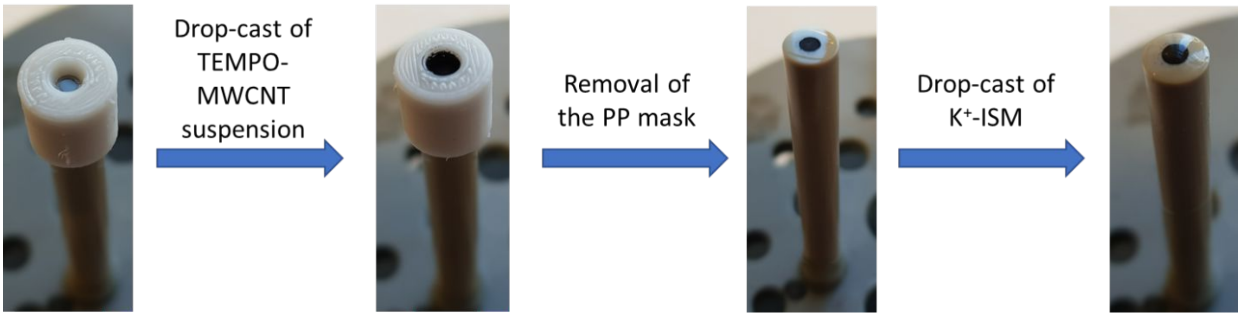

Scheme S1. Fabrication procedure of the TEMPO-MWCNT based K^+-SCISE

Characterization of the TEMPO-MWCNT solid contact with cyclic voltammetry

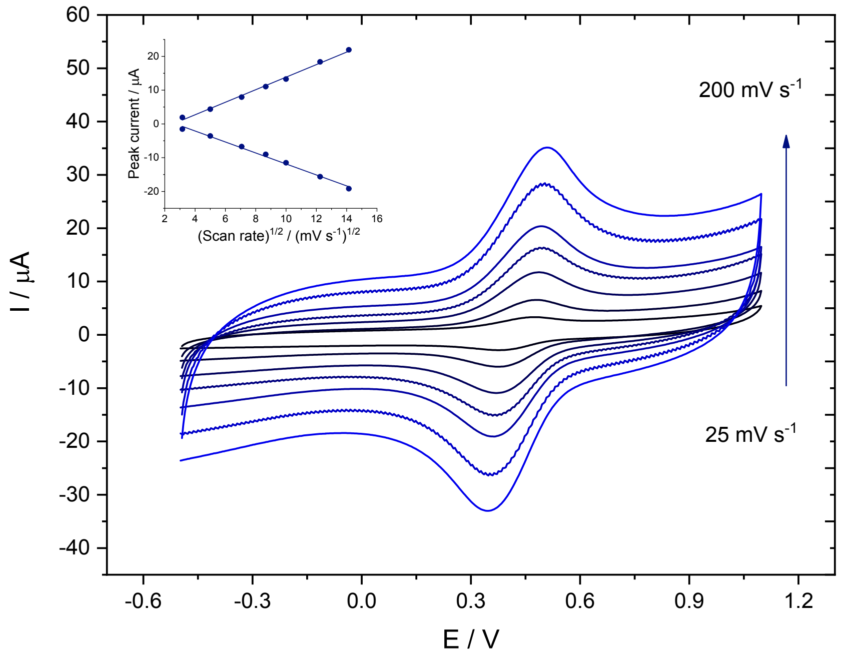

Figure S1. Cyclic voltammetric curves of the TEMPO-MWCNT solid contact with different scan rates (25 – 200 mVs^{-1}) measured in 0.1 M $TBAPF_6$ in acetonitrile. 20 μL of 5 mg/mL TEMPO-MWCNT suspension in THF was drop-cast onto the glassy carbon surface. The inset shows the peak currents vs. the square root of the scan rate. RE: Ag/0.01 M $AgNO_3$//0.1 M $TEANO_3$, CE: Pt.

Chronopotentiometric characterization of TEMPO-MWCNT based K^+-SCISE and coated-wire ISE

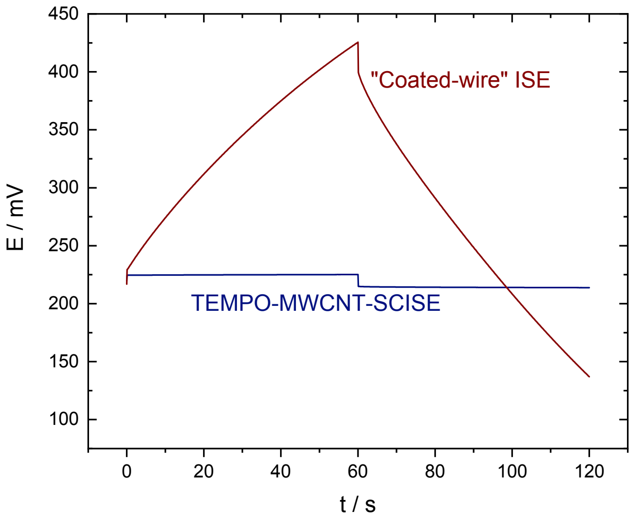

Figure S2. Chronopotentiograms of TEMPO-MWCNT based K^+-SCISEs (blue line) and coated-wire K^+-ISE (red line), measured in 0.01 M KCl. Applied current: $+10^{-9}$ A for the first 60 s and -10^{-9} A for the next 60 s. RE: Ag/AgCl, CE: Pt.

As expected, the capacitance of the TEMPO-MWCNT based SCISE (95.3 µF) is much higher than that of the coated-wire electrode (0.27 µF). The capacitance of the TEMPO-MWCNT layer before coverage (597.1 µF) determined by EIS in acetonitrile solution is ca. 6 times larger than that of TEMPO-MWCNT-based K^+-SCISEs. This is most likely caused by the limited interpenetration of the solid contact layer with the ion-selective membrane compared to the acetonitrile solution (0.1 M tetrabutylammonium hexafluorophosphate solution). It is common that the capacitance of thicker, uncovered solid contacts is larger than of the relevant membrane coated SCISE[1,2].

Table S1. Calibration parameters for two fabrication batches of TEMPO-MWCNT based K^+-SCISEs (n = 5 – 5 electrodes). The electrodes were short-circuited with each other for 48 hours in 0.01 M KCl prior to the calibration.

	1st Batch (n = 5)		2nd Batch (n = 5)		Interbatch	
	Average	Std. dev.	Average	Std. dev.	Average	Std. dev.
E^0 (mV)	340.9	0.62	375.2	0.69	358.1	18.10
Slope (mV/dec)	58.4	0.2	59.0	0.1	58.7	0.32

Potentiometric selectivity coefficients of TEMPO-MWCNT-based K^+-SCISEs

The potentiometric selectivity coefficients of TEMPO-MWCNT-based K^+-SCISEs were determined with the separate solution method. Potentials were measured in 0.1, 0.01 and 0.001 M chloride salts of K^+, and the interfering ions Li^+, Na^+, NH_4^+ and Mg^{2+}. RE: Ag/AgCl/3 M KCl//1 M LiOAc.

Table S2. Potentiometric selectivity coefficients of TEMPO-MWCNT based K^+-SCISEs (n = 3)

X	Log K
**Li^+**	-4.55±0.05
**Na^+**	-4.11±0.08
**Mg^{2+}**	-4.45±0.00
**NH_4^+**	-1.85±0.00

Measurements of K^+ in serum

To investigate the applicability of TEMPO-MWCNT-based K^+-SCISEs in meaningful complex, biological samples, we tested them for the assessment of K^+ in serum samples. The measurements were performed in undiluted serum samples with 3 identically prepared K^+-SCISEs. The concentration of K^+ in the serum sample was confirmed by Flame – Atomic emission spectroscopy (F-AES) using acetylene – air flame and 50-fold diluted serum. The electrodes were first tested by calibration in standard

solutions (Table S3. Cal1-Cal4) with constant ionic strength (160 mM) that revealed Nernstian response (Fig. S3A).

Table S3. The composition of standard solutions for calibration.

Standard solutions	c (Na^{+}) / mM	c (K^{+}) / mM	c (Ca^{2+}) / mM
Cal1	156.5	0.5	1
Cal2	156	1	1
Cal3	154.5	2.5	1
Cal4	152	5	1
Cal5	155	2	1
Cal6	152.6	4.4	1
Cal7	149	8	1

Next, the potential of the electrodes was recorded in 10.0 mL of undiluted human serum and the sample was spiked with 150 µL potassium standard solution (0.1 M). As shown in Fig. S3 B, the ISEs have a reproducible and stable potentiometric response in the serum sample already within ca. 1 min. The potassium ion concentration of the human serum sample was calculated using the standard addition method. The average concentration value determined with 3 electrodes was 1.94 ± 0.02 mM. The potassium concentration of the serum was confirmed by Flame Atomic Emission Spectroscopy (2.1 mM).

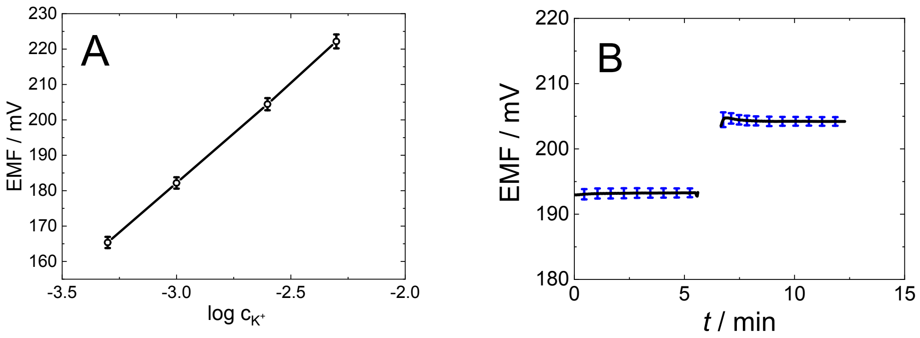

Figure S3. (A) Potentiometric calibration curve of TEMPO-MWCNT-based K^{+}-SCISEs (n=3) for potassium ion using standard solutions of constant ionic strength mimicking the ionic content of serum. (B) Relevant potential traces in undiluted human serum sample in a typical standard addition measurement.

"Calibration-free" measurements of K^+ concentration in serum

For a preliminary testing of the opportunities for calibration-free measurements of K^+ concentrations in undiluted serum a single randomly chosen electrode from a fabrication batch of 4 electrodes was calibrated in standard solutions (Table S3 Cal2 and Cal5-Cal7) and then this calibration (Fig. S4A) was used to determine the concentration of a serum sample with a nominal concentration of 4.4 mM (Normal Human Serum, Merck Millipore, Lot: 3733758). The determined K^+ concentration was 4.1 ± 0.1 mM, with a deviation of ca. 7 % from the value provided by the supplier.

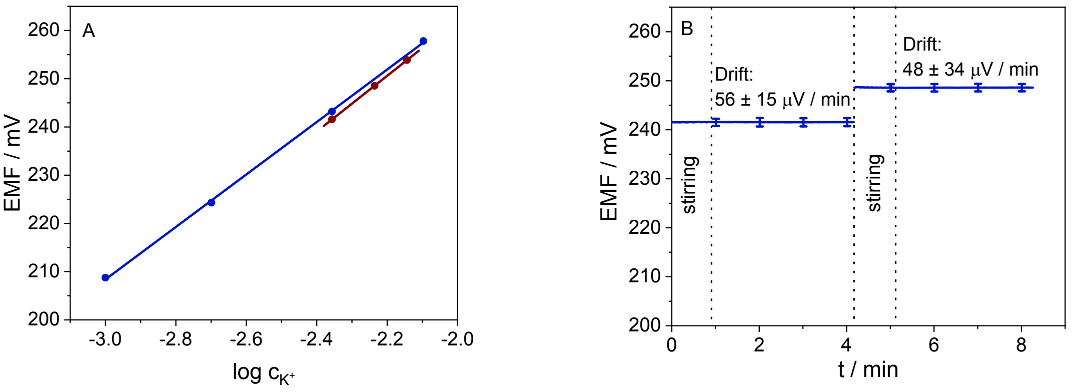

Fig S4. (A) Potentiometric calibration curves of TEMPO-MWCNT-based K^+-SCISE for potassium ion. The blue line shows a calibration curve obtained when solutions of constant ionic strength were used mimicking the ionic content of serum. The red line shows the calibration taken in human serum with known potassium ion concentration. (B) Relevant potential traces in undiluted human serum sample in a typical standard addition measurement (n = 4).

To further reduce this already small deviation, which may be due to components of the serum affecting the electrodes, we performed the calibration using as a background a pooled serum (10 mL) with accurate potassium concentration that was spiked with aliquots of 150 µL concentrated KCl solutions (0.1 M) (Fig S4A red). Repeating the same experiment as before using a new batch of freshly prepared SCISEs (n=4) the calculated potassium ion concentration was 4.4 ± 0.2 mM, practically identical to that given by the supplier (4.4 mM). The relative error of each SCISE in this batch was lower than 5%. This matches in terms of potassium ion measurement the specifications of the i-STAT system, which is a commercial single use portable device.[3]

References:

(1) Bobacka, J. Potential Stability of All-Solid-State Ion-Selective Electrodes Using Conducting Polymers as Ion-to-Electron Transducers, *Anal. Chem.* **1999**, *71*, 4932-4937.
(2) Szűcs, J.; Lindfors, T.; Bobacka, J.; Gyurcsányi, R. E. Ion-selective Electrodes with 3D Nanostructured Conducting Polymer Solid Contact, *Electroanal.* **2016**, *28*, 778-786.

(3) Mock, T.; Morrison, D.; Yatscoff, R. Evaluation of the i-STAT™ system: A portable chemistry analyzer for the measurement of sodium, potassium, chloride, urea, glucose, and hematocrit, *Clin. Biochem.* **1995**, *28*, 187-192.
